# Alkaloid Escholidine and Its Interaction with DNA Structures

**DOI:** 10.3390/biology10121225

**Published:** 2021-11-24

**Authors:** Petra Jarošová, Pavel Hannig, Kateřina Kolková, Stefania Mazzini, Eva Táborská, Raimundo Gargallo, Gigliola Borgonovo, Roberto Artali, Petr Táborský

**Affiliations:** 1 Department of Chemistry, Faculty of Science, Masaryk University, Kamenice 5, 62500 Brno, Czech Republic; petrajarosova1@gmail.com (P.J.); 461195@mail.muni.cz (P.H.); 474269@mail.muni.cz (K.K.); 2 Department of Food, Environmental and Nutritional Sciences (DEFENS), Section of Chemical and Biomolecular Sciences, University of Milan, Via Celoria 2, 20133 Milan, Italy; stefania.mazzini@unimi.it (S.M.); gigliola.borgonovo@unimi.it (G.B.); 3 Department of Biochemistry, Faculty of Medicine, Masaryk University, Kamenice 5, 62500 Brno, Czech Republic; taborska@med.muni.cz; 4 Department of Chemical Engineering and Analytical Chemistry, University of Barcelona, Marti i Franquès 1, E-08028 Barcelona, Spain; raimon_gargallo@ub.edu; 5 Scientia Advice di Roberto Artali, 20832 Desio, Italy; roberto.artali@scientia-advice.com

**Keywords:** escholidine, G-quadruplex, DNA, cancer, alkaloid, spectroscopy

## Abstract

**Simple Summary:**

Escholidine is a rare protoberberine alkaloid present in trace amounts in roots of *Eschscholtzia californica* and in the aerial parts of *Hunnemannia fumariaefolia*. Due to the characteristic charged structure, it can interact with various forms of nucleic acids, including non-canonical structures. A series of spectroscopic experiments have shown notable melting stabilization of antiparallel G-quadruplex sequence DL40 induced by escholidine (ΔT_m_ = 5.2 °C). Interaction stoichiometry calculated from fluorescence titration curves was estimated to be 4:1 or 5:1 (alkaloid:DNA). Nuclear Magnetic Resonance (NMR) experiments have confirmed that an external loop binding is likely responsible for this stabilization. The three-dimensional model of the complex between escholidine and DL40, obtained as a result of the molecular docking experiment, implies the preferred orientation of escholidine to the quadruplex structure. Since the stabilization of telomeric G-quadruplex structures by small ligands is often used as a strategy in anti-cancer therapy, alkaloid escholidine seems to be an interesting agent from a medicinal point of view.

**Abstract:**

Berberine, the most known quaternary protoberberine alkaloid (QPA), has been reported to inhibit the SIK3 protein connected with breast cancer. Berberine also appears to reduce the bcl-2 and XIAP expression-proteins responsible for the inhibition of apoptosis. As some problems in the therapy with berberine arose, we studied the DNA binding properties of escholidine, another QPA alkaloid. CD, fluorescence, and NMR examined models of i-motif and G-quadruplex sequences present in the n-myc gene and the c-kit gene. We provide evidence that escholidine does not induce stabilization of the i-motif sequences, while the interaction with G-quadruplex structures appears to be more significant.

## 1. Introduction

Quaternary protoberberine alkaloids (QPA) represent a large class of isoquinoline alkaloids [1,2,3,4]. They consist of variations of a tetracyclic ring system, a derivate of 5,6-dihydrodibenzo[*a*,*g*]quinolizinium [5,6,7]. The most famous QPA is berberine, which is used to treat, for example, diabetes and hypercholesterolemia in traditional medicine [8]. Moreover, berberine is examined as a potential treatment for breast cancer. Expression of the gene coding protein SIK3 is connected with breast cancer, and berberine combined with emodin can inhibit this protein [9]. In vivo study proved that berberine reduced bcl-2 and XIAP expression-proteins responsible for inhibition of apoptosis [10]. Other studies interested in treating colon cancer [11] demonstrated the positive effect of berberine. However, some berberine properties, as induction of drug resistance, can be a problem for the therapy [8]. The problem could be solved by combining different treatments or finding other alkaloids with similar properties within the class of QPA. A deeper study of developments in cells can be helpful.

**Figure 1:**
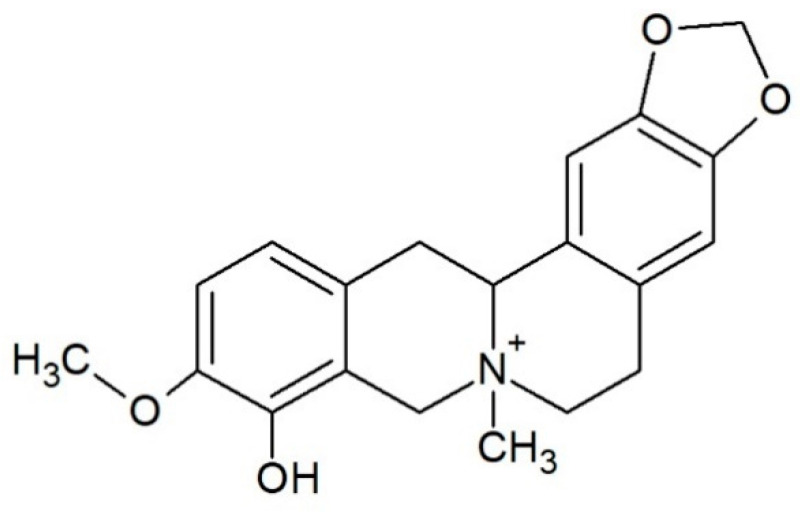
Structure of alkaloid escholidine.

Escholidine (Figure 1) is a QPA [12] present in the plant *Escholtzia californica* [13]. It is used as a sedative and painkiller, especially useful for treating sleep and dream-related disorders served in the form of teas, tinctures, or extracts [14,15]. In the plant other alkaloids are also present, such as californidine and escholtzine, which are the major ones, as well as sanguinarine, sanguirubine, or macarpine [16].

Recent studies highlight the potential importance of non-canonical forms of DNA, such as G-quadruplex (G4) or i-motif, in replication, transcription, or translation processes and mRNA splicing [17,18,19]. Non-canonical DNA structures are characterized by their specific sequence. G-quadruplexes are formed by guanine-rich sequences and stabilized by cations, such as K^+^ or Na^+^ [20]. The i-motif structures are formed by cytosine-rich sequences and are stable at slightly acid pH values. Both structures are found at the end of telomers and near the promoter region of several oncogenes such as *bcl-2*, *c-myc*, VEGF and RET [19].

**Figure 2:**
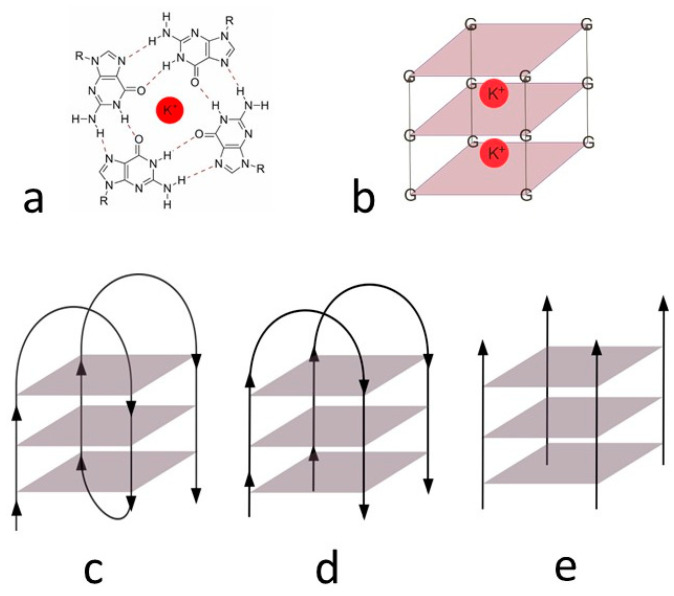
G-tetrad (**a**) and three-dimensional schematic structure with potassium ions (**b**), unimolecular (**c**), bimolecular, (**d**) tetramolecular, and (**e**) G-quadruplex.

The elementary unit of G4 is the “π-stacked” guanine tetrad, also called the G-quartet, which forms an almost planar structure. This tetrad consists of four cyclically arranged guanine bases rotated 90° and connected by a total of eight Hoogsteen hydrogen bonds linking amino and imino protons of one base to nitrogen N7 and oxygen O6 atoms of the adjacent base, respectively (Figure 2a,b). G-quartets are layered on top of each other in a spatial arrangement. There is a free space in the center of the guanine tetrads, which is filled mainly by univalent alkali metal cations (K^+^, Na^+^) [21,22,23,24].

The G-quadruplex structures can consist of several strands of different molecules or be formed intramolecularly. Figure 2 shows unimolecular (c), bimolecular (d), and tetramolecular (e) type. Furthermore, fibers of the G-quadruplex can be arranged in a variate direction. They can form parallel G-quadruplex, otherwise antiparallel. The most common type, which is present in living cells, is the unimolecular G-quadruplex. There are also hybrid structures containing three fibers in the same direction and one in the opposite direction [25].

**Figure 3:**
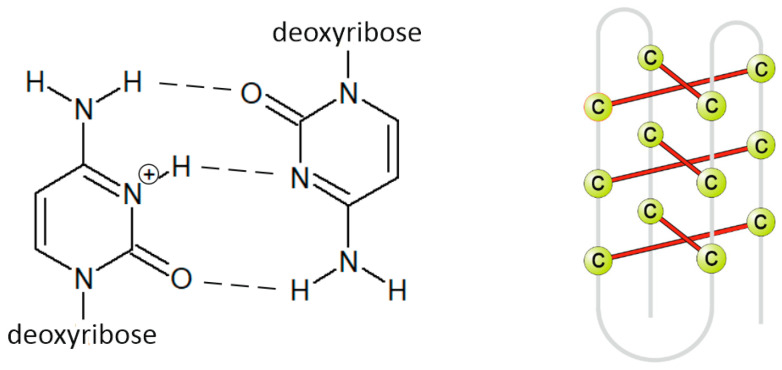
Primary (**left**) and secondary (**right**) structure of i-motif.

The i-motif consists of two parallel duplexes zipped together by the intercalation, cytosines bonds together by three hydrogen bonds (Figure 3). One of the cytosines must be in a protonated form to form hydrogen bonds, hence the reason why i-motifs are so sensitive to pH [26]. They exist rather in acidic solution [27,28,29] because at pH higher than 6.5, approximately, cytosine bases are deprotonated, and hydrogen bonds disappear. The exception is i-motives with a tract length of at least five cytosines, which can also exist in physiological pH [30]. In addition to pH, the temperature and length, and structure of the loop also affect the stability of i-motif [31,32]. Due to the influence of pH on its stability, the i-motif structure has not received so much attention in the past because it was assumed that it could not occur in a physiological environment. The interest in i-motif has grown in recent years because it has been proved that i-motives can occur in vivo and in the presence of an acidic microenvironment in some tumor cells (pH of 6.5–6.8) [33,34,35,36,37,38].

In this work, we study the influence of alkaloid escholidine on the stability of a set of DNA sequences forming different G-quadruplex and i-motif structures. While increased stability of G-quadruplex:ligand complex over the G-quadruplex alone was proven with many groups of chemicals (protoberberine [39] and benzo[c]phenanthridine [40] alkaloids, antraquinones [41]), increased stability of i-motif with ligand was observed only in several cases [42]. In addition, berberine was found to bind an AT-rich sequence in the minor groove of the double helix [43], while no specific interactions with any C-rich sequence were found [44].

The sequence 5′-CG_3_CG3CGCGAG3AG4-3′ (ckit21) is part of the *c-kit* gene that encodes receptor tyrosine kinase. A stable parallel G-quadruplex structure was identified on this sequence in humans, chimpanzees, and rats [45]. Sequence 5′-G_3_CG3CGCGAG3AG3T-3′ (GG1) was prepared by removing the first cytosine and G to T mutation at position 21. A further G to T mutation at position 12 was also considered (ckit21T12T21) [46].

The sequence 5′-G2T4G2CAG3T4G2T-3′ (DL40) forms a unimolecular antiparallel G-quadruplex, containing two diagonal loops and one reversal loop [46].

The sequence 5′-AC5TGCATCTGCATGC5TC3AC5T-3′ (nmyc01), located near the promoter region of the *n-myc* gene, presents an i-motif conformation. An increased presence of this *n-myc* gene is associated with an increased incidence of tumors, mainly neuroblastoma. In this work, we studied the interactions of escholidine with this sequence and with the TGCA to TTTT mutated sequences at 13–16 level (nmyc01m) to prevent hairpin structure formation [47,48].

The sequence 5′-C3TA2C3TA2C3TA2C3T-3′ (22nt) contains sequence CCCTAA, which frequently occurs in telomeric DNA. In lower eukaryotes presence of G-quadruplexes was confirmed. Telomeres play an important role in cancer; thus are strictly regulated in human cells. Cancer cells often secrete many telomeres leading to cancer growth [23,49,50].

## 2. Materials and Methods

Alkaloids were extracted from plant material in the Department of Biochemistry, Faculty of Science, Masaryk University (Brno, Czech Republic). The oligonucleotides used in this work (Table 1) were purchased as dry samples from Thermo Fisher Scientific (Waltham, MA, USA) at HPLC grade. Other reagents, such as KH_2_PO4, KCl, NaOH, KOH, and CH_3_COONa (all p.a. grade), were purchased from Lach-Ner (Neratovice, Czech Republic).

### 2.1. Melting Experiments

The melting temperature (T_m_) of oligonucleotides was determined by CD spectroscopy on a Jasco J815 device. For all measurements, samples were prepared with a final concentration of 2 µM oligonucleotide and 8 µM alkaloid. G-quadruplexes were prepared in phosphate buffer solution (5 mM KCl, 10 mM KH_2_PO_4_ pH 7). The i-motif samples were prepared in acetate buffer (0.2 M CH_3_COONa, pH 5).

A magnetic stirrer and temperature sensor were inserted into the cuvette. Spectra of ellipticity dependence on wavelength from 220 to 330 nm were measured between 20–95 °C for each sample. The wavelength at which the signal reaches the maximum (nmyc01, nmyc01m, 22nt at 287 nm, DL40 at 291 nm, GG1 at 261 nm) was deducted from the measured spectra. During further measurements, ellipticity was measured only at the selected wavelength with ΔT = 0.2 °C with increasing/decreasing temperature 1 °C/min. The sample was measured in both directions (heating and cooling) to determine the potential presence of hysteresis. The ellipticity/temperature dependence graph has been created from the selected wavelength. From this graph, thermodynamic variables, such as melting temperatures and enthalpy and entropy changes, were calculated using an in-house-made program written in Matlab^®^. The algorithm was based on that which was previously published by Puglisi [51] and Breslauer [52], using a two-state unfolding process and considering the changes in enthalpy and entropy invariable in the temperature range studied. Graphs were smoothed by the Savitzky–Golay function.

### 2.2. Fluorescence Titrations

Alkaloid solution was placed into a cuvette, the concentration of escholidine was 3 × 10^−5^ mol∙L^−1^. The solution also contained KCl at a concentration of 0.005 mol∙L^−1^ and a buffer of 0.01 mol∙L^−1^, phosphate buffer pH 7 for structures of G-quadruplex, acetate buffer pH 5 for i-motif structures. A titrating solution of oligonucleotide was prepared and also contained an alkaloid, KCl, and a buffer of the same concentration as in the alkaloid solution, not changing their concentration in the cuvette.

The fluorescence spectrum of the pure alkaloid solution was measured first. Then, the alkaloid solution was titrated with the oligonucleotide solution, and the fluorescence spectrum was measured after each addition.

All spectra were transferred to the Matlab^®^ program, where they were fitted to curves corresponding to individual stoichiometric ratios using the Equispec function [53]. The best model was selected by the least-squares method to determine the stoichiometric ratio of the DNA:ligand complex, and the association constant log K_a_ was obtained.

### 2.3. NMR Spectroscopy

NMR spectra were recorded on a Bruker AV600 spectrometer operating at a frequency of 600.10 MHz, equipped with a 5 mm TXI inverse probe and *z*-axis gradients. The ^1^H spectra were acquired at 25 °C. The NMR sample of i-motif oligonucleotides (nmyc01, nmyc01m, and 22nt) was prepared in H_2_O/D_2_O (9:1) concentration range from 0.4 to 0.9 mM containing sodium phosphate buffer, pH 5.0. The G-quadruplex DL40 was prepared in H_2_O/D_2_O (9:1) in buffer containing 150 mM NaCl and sodium phosphate pH 6.8. The sample of c-kit21was prepared at a concentration of 0.40 mM, dissolved in 5 mM KH_2_PO_4_, 20 mM KCl, pH 6.9. Moreover, for the NMR studies, also the c-kit21T12T21-mer sequence, 5′- CGGGCGGGCGCT**^12^**AGGGAGGGT**^21^**-3′ was used as the G to T mutation at position 12, and 21 further stabilizes, at low KCl concentration, the monomeric intramolecular parallel G-quadruplex [54].

The samples were heated to 85 °C for 1 min and then cooled at room temperature overnight. The 2D NOESY spectra of DL40 were performed using standard pulse sequences in phase-sensitive mode with 300 ms of mixing time. Raw data were Fourier-transformed after apodization with a 90°-shifted sine-bell-squared function, zero-filling to 2 K × 2 K real data points, and baseline corrected. The NMR data were processed using TOPSPIN 1.3 and analyzed by the Sparky program (University of California, San Francisco, CA, USA). Small amounts of the escholidine stock solution in DMSO-*d*_6_ (52 mM) were added at different R = (drug)/(DNA) ratios to the oligonucleotide solutions.

### 2.4. Molecular Modeling Studies

We used docking calculations to elucidate interaction model [55]. The starting three-dimensional structure of escholidine was obtained from PubChem (PubChem CID: 102387713) [56], while that of the antiparallel G-quadruplex DL40 was taken from the NMR ensemble deposited in the Protein Data Bank (PDB accession code: 1i34) [46].

Flexible docking calculations at the DL40 target were performed by AutoDock 4.2 [57], using the Lamarckian Genetic Algorithm in combination with a grid-based energy evaluation method was to calculate grid maps (80 Å × 80 Å × 80 Å box with a spacing of 0.01 Å). Gasteiger–Marsili charges [58] were added to the ligand by using AutoDock Toolkit (ADT) [59]. The solvation parameters were added to the system by means of the Addsol utility of AutoDock, and the phosphorus atoms in the G-quadruplex structure were parameterized using the Cornell parameters. The experiment was conducted with an initial population consisting in 100 randomly placed escholidine molecules. The maximum number of energy evaluations was set at 250 with an elitism value of 1, a mutation rate of 0.02 and a crossover rate of 0.80. The local search was conducted using 250 independent docking runs using the pseudo-Solis and Wets algorithm, with a maximum of 250 iterations per local search. The docking results were scored by using an in-house version of the simpler intermolecular energy function based on the Weiner force field, and the lowest energy conformations (differing by less than 1.0 Å in positional root-mean-square deviation (rmsd)) were collected.

Molecular graphics and analyses performed with UCSF ChimeraX, developed by the Resource for Biocomputing, Visualization, and Informatics at the University of California, San Francisco, with support from National Institutes of Health R01-GM129325 and the Office of Cyber Infrastructure and Computational Biology, National Institute of Allergy and Infectious Diseases [60].

## 3. Results and Discussion

### 3.1. CD Spectra and Meltings

First, CD spectra of the considered oligonucleotides were measured in the absence and presence of escholidine.

**Figure 4:**
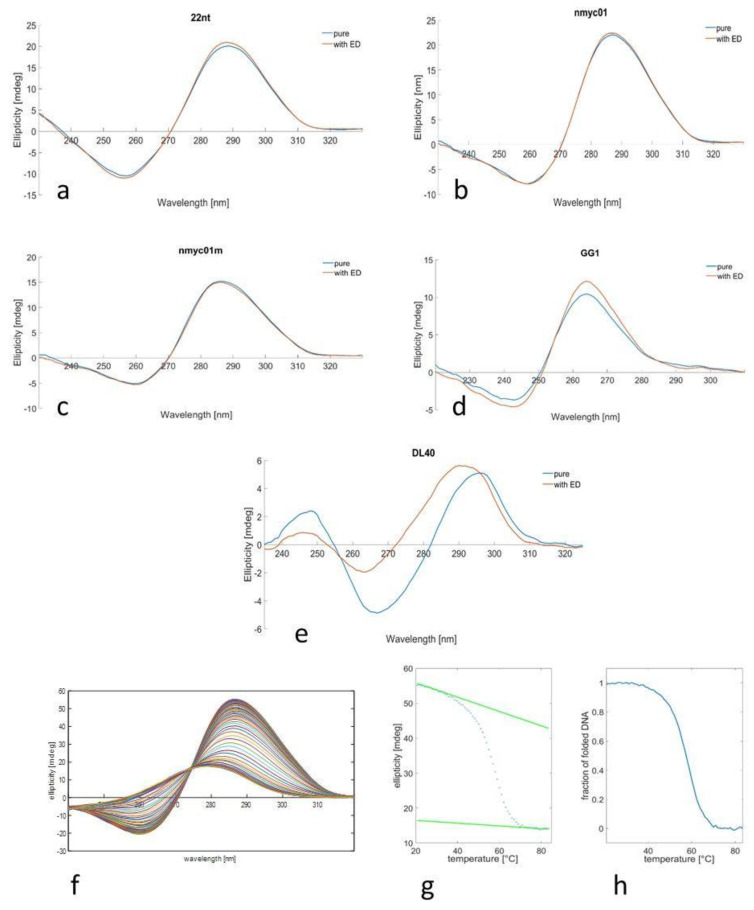
pH 5: CD spectra of 22nt (**a**), nmyc01 (**b**), and nmyc01m (**c**) in the absence (blue) and presence (red) of escholidine in acetic buffer (c_oligo_ = 2 µM, t = 20 °C). pH 7: CD spectra of GG1 (**d**), and DL40 (**e**), in the absence (blue) and presence (red) of escholidine at phosphate buffer (coligo = 2 µM, t = 20 °C). Temperature dependence: CD spectra recorded along with the melting experiment of nmyc01 (**f**), ellipticity trace measured at 287 nm (**g**), and the fraction of folded DNA calculated assuming an intramolecular two-step process (**h**).

Oligonucleotides 22nt, nmyc01, and nmyc01m form i-motif structures at slightly acidic pH values. Hence, at pH 5 the spectra of the three nucleotides have the same shape with a positive peak around 290 nm and a smaller negative peak around 260 nm, which are indicative of the formation of i-motif structures. There was no apparent effect of escholidine on the shape and intensity of the spectrum of any of these sequences (Figure 4), indicating that the potential interaction with the drug did not modify the overall i-motif structure.

A similar procedure was carried out for guanine-rich sequences (Figure 4). The GG1 oligonucleotide forms a parallel G-quadruplex structure, as denoted by the position of the main CD bands: one positive around 260 nm and one negative around 240 nm [35]. The shape of the spectrum of the oligonucleotide alone and with escholidine is the same, only slightly different in intensity. On the other hand, the DL40 oligonucleotide shows a CD spectrum with different features: an intense positive peak around 295 nm, a negative peak around 265 nm, and a small positive peak around 245 nm [61] are characteristics of antiparallel G-quadruplex sequence. Upon the ligand, evident changes were observed in the CD spectrum, suggesting a specific drug interaction with the G-quadruplex that produced some structural modifications.

CD-monitored melting experiments in the absence and presence of the drug were carried out to gain insight into the potential stabilization of the structures upon interaction with escholidine. Figure 4 shows an example of this procedure.

Table 2 summarizes the melting temperature (Tm) of the DNA structures in the absence and presence of escholidine. In all cases, a two-stage process was considered. This assumption was checked by multivariate analysis of the whole set of spectra measured along with each melting experiment.

The most stable structure in terms of the T_m_ value was the G4 structure DL40. In the presence of escholidine (1:4 DNA:ligand ratio), some variations in T_m_ values were observed. In the case of nmyc01, thermal stability was not increased as in the case of the mutated version of this oligonucleotide (nmyc01m). Additionally, minimal stabilization was observed in the case of the i-motif formed by 22nt, which does not include any Watson–Crick base pair. Overall, this suggests that escholidine does not interact strongly with the i-motif core, i.e., C·C^+^ base pairs that stabilize the structure.

In the case of the parallel G-quadruplex structure formed by GG1, a slight increase was observed. However, the most significant change can be observed with antiparallel DL40 (ΔT_m_ = 5.2 °C), which agrees with the evident changes observed in the CD spectrum upon adding the ligand.

The thermodynamic analysis allowed the determination of the changes in enthalpy, entropy, and Gibbs free energy associated with each transition, assuming a two-stage process and a constant value of the changes in enthalpy and entropy (Table 3). Considering the uncertainties associated with the determined values of the thermodynamic parameters, the interaction of escholidine with the considered sequences produces minor variations.

### 3.2. Fluorescence Titrations

Fluorescence measurements were performed to determine binding stoichiometries and association constants between escholidine and various oligonucleotide structures. Of the titration, the fluorescence spectra of alkaloid without oligonucleotide were measured. The emission maximum was found to be 330 nm. A wavelength of 285 nm was used for the excitation of samples. When DNA was added, the fluorescence intensity at 330 nm decreased. The fluorescence quenching induced by DNA to alkaloid interaction enabled us to calculate stability constants and stoichiometry. Examples of recorded spectra and titration curves are shown in Supplementary Materials. Subsequently, Equispec tool was used for data treatment. Each stoichiometry model was considered according to the smallest sum of squares (Supplementary Materials).

The best-fitting model was usually 4:1 or 5:1 (alkaloid:DNA). Thus, the interaction and stabilization of structures are likely to occur through multiple binding mechanisms at once. The stoichiometry models, as well as the calculated association constants, are collected in Table 4. When the complexity of the models is omitted, association constants are similar for all studied structures.

### 3.3. NMR Analysis

Imino protons in the double helix, i-motif, and G-quadruplex structures of DNA are involved in different types of hydrogen bonds, and their chemical shifts in ^1^H NMR spectra are characteristic for each structure: 12–14 ppm for Watson–Crick CG and AT base pairs, 15–16 ppm for C-C^+^ base pairs and 10–12 ppm for G-quartets.

**Figure 5:**
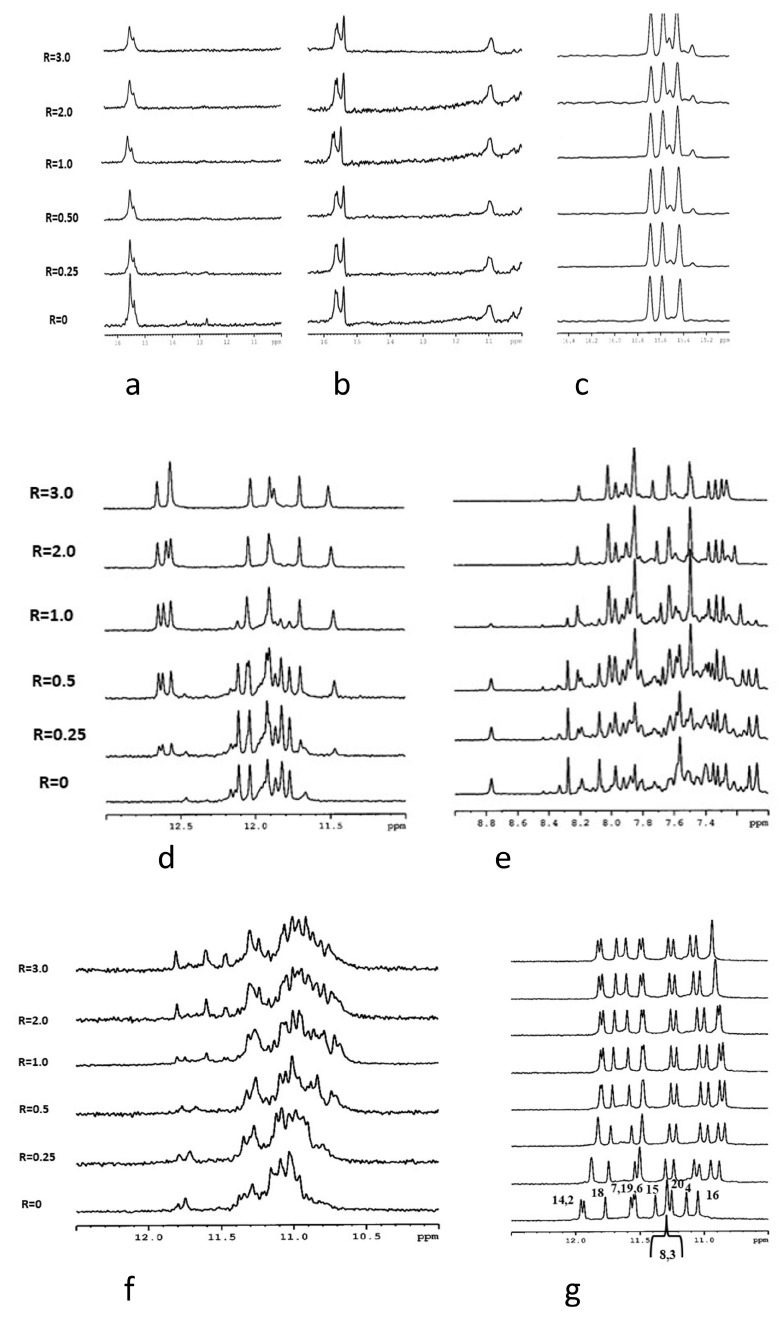
Imino proton region of the 1D NMR titration spectra of nmyc01 (**a**), nmyc01m (**b**), and 22nt (**c**) at 25 °C and at different R = [drug]/[DNA] ratios. Imino protons (**d**) and aromatic protons (**e**) regions of the 1D NMR titration spectra of DL40 at 25 °C and different R = (drug)/(DNA) ratios. Imino protons region of the 1D NMR titration spectra of GG1 (**f**) and ckit21T12T21 (**g**) at 25 °C and different R = (drug)/(DNA) ratios.

The spectra of nmyc01, nmyc01m, and 22nt (nucleotide alone) present signals at 15–16 ppm, showing the formation of i-motif structures (Figure 5). Some differences between nmyc01 and the mutated sequence nmyc01m can be observed. The spectrum of nmyc01 (Figure 5a) showed very weak signals between 12.5–14 ppm, attributable to Watson–Crick base pairs formation in the TGCA tract, which is not present in the mutated sequence nmyc01m. The spectrum of nmyco1m (Figure 5b) showed intense signals at 11 ppm, assigned to T·T base pair formation, due to the presence of the TTTT tract that has replaced the TGCA fragment. These base pairs are common in the loop residues connecting C-tracts in i-motif structures [62].

The titration experiment of nmyc01 revealed a slight line broadening of the signals at 15–16 ppm, due to the C-C^+^ NH proton pairs, while the weak signals at 12.5–14 ppm decreased and disappeared for R = (ligand)/(DNA = 0.25) (Figure 5a). This suggests some interaction of escholidine to the i-motif, with the disruption of the Watson–Crick base pairs of the hairpin structure. The same experiment with the mutated sequence cmyc01m did not cause any change in the resonances of the imino protons, suggesting that escholidine neither intercalated nor stacked externally to the cytosine bases (Figure 5b). Upon addition of escholidine to 22nt oligonucleotide, only small insignificant changes in the imino protons of hemiprotonated cytosines occurred (Figure 5c).

The structural polymorphism of G-quadruplex structures makes them versatile targets of ligands, consequently stabilizing them. As the interaction of escholidine with G quadruplexes seems to be more promising from CD results, we chose the DL40 for NMR experiments. The spectrum of this oligonucleotide alone showed in our experimental conditions both sharp and broad NH imino signals (Figure 5d). The same behavior is shown by the non-exchangeable aromatic resonances, indicating the conformational heterogeneity of the G-quadruplex (Figure 5e). Upon each addition of escholidine, remarkable changes in the NMR spectra were observed, showing evidence of some interaction. Even at low R = 0.25, a new set of imino signals associated with the guanine moieties of the tetrad at *δ* = 12.5–12.8 ppm is observed. All the other signals, including the aromatic ones, still appear very crowded at this R ratio. At R ≥ 1.0, the imino and the aromatic protons region became less complicated, indicating a single folding conformation; thus, some stabilization of the G-quadruplex structure due to the ligand must occur. The resonances of escholidine are too broad and could not be detected. Their broad shape indicated that the ligand can either move in the binding site or bind to different binding sites of the target. For this reason, we decided to examine the ckit2 21 mer, which shows a parallel G-quadruplex structure in comparison with the antiparallel structure of DL40. This nucleotide adopts in K^+^ solution two distinct antiparallel G-quadruplex conformations in slow exchange, forming either a monomeric or a dimeric structure, depending on the concentration of KCl [29,30]. Unfortunately, the simultaneous presence of the two species causes a line widening in the signals (Figure 5a, R = 0). The addition to escholidine slightly improves the appearance of the spectra, but the binding with the ligand is not sufficient to stabilize one of the two conformations. Thus, the NH imino signals remain very broad (Figure 5f).

**Figure 6:**
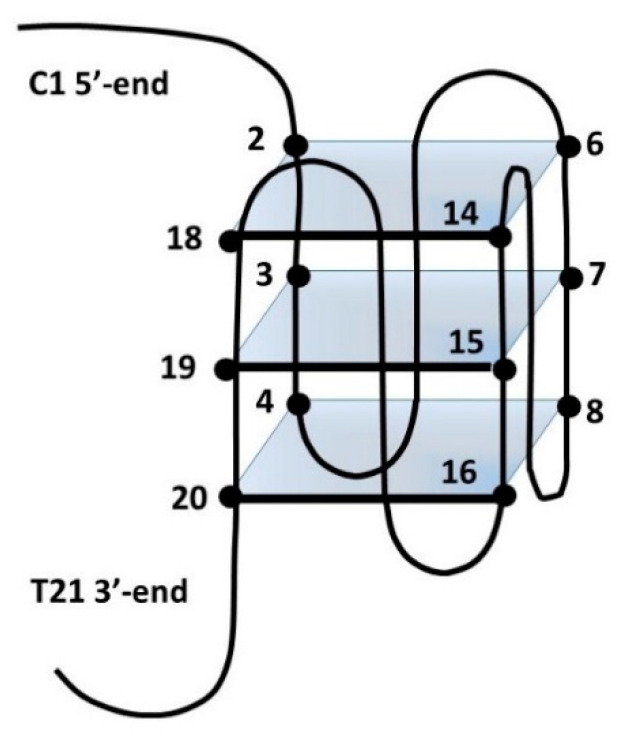
Schematic representation of ckit21T12T21. Numbers refer to the position of bases within this sequence.

It was found that the G to T mutation at positions 12 and 21 of the above sequence further stabilizes, at low KCl concentration, the monomeric intramolecular parallel G-quadruplex (ckit2T12T21) [45,54]. In these conditions, the NH imino protons are well resolved, and the addition of escholidine caused changes in the NMR spectrum, even at a low R ratio = 0.25/1.0 (Figure 5g). During the titration experiment, the presence of the imino protons in 10.8–12.0 ppm indicated that the G-quadruplex structure was maintained. This is confirmed by the assignments of the guanine NH and H8 protons, performed through the sequential NOEs interactions of the imino signals and the inter-residue NOE connectivity between NH and H8 resonances, characteristic of the three tetrads (Table S1). The non-exchangeable proton signals also sharpened, whereas those of escholidine remain very broad and could not be assigned. By increasing the R until a value of 3, the spectrum did not change significantly. Most of the NH signals of the external tetrads moved to upfield, but such effect is not equally distributed over the four guanines of each tetrad. Specifically: G2 and G18 NH (Δδ −0.19 ppm and −0.14 ppm) at the 5′-terminal end, G4 NH (Δδ −0.22 ppm) G20 (Δδ −0.18 ppm) and G8 (Δδ −0.26 ppm) at 3′-end (Figure 6 and Table S2). These results indicate that escholidine interacts with the G-quadruplex positioning over the external tetrads but covering only half of the guanine planes. It appears preferentially located over the guanines G2, G18 of the 5′-end, and over the guanines G4 at 3′-end.

### 3.4. Molecular Modeling Studies

**Figure 7:**
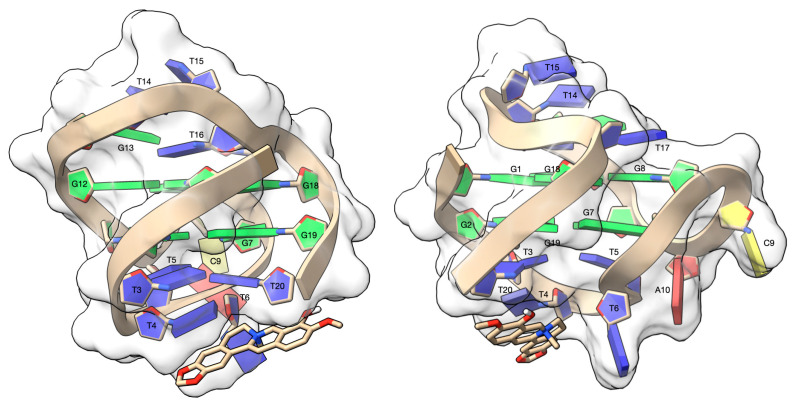
Side views of the complex between escholidine and the DL40 G-quadruplex, as obtained from the molecular docking experiment. The G-quadruplex target is contoured by a ghostly-white solvent accessible surface (SAS) and the nucleotides were depicted as filled rings: adenine in red, cytosine in yellow, guanine in green and thymine in blue. The escholidine alkaloid is represented as van der Waals (vdW) spheres and coloured according to the atom types. The drawing was created by using the Chimera-X software (Resource for Biocomputing, Visualization, and Informatics San Francisco, CA, USA) [63].

The three-dimensional model of the complex between escholidine and DL40, as obtained from the blind molecular docking experiment, is shown in the Figure 7.

No counterions were added to the starting three-dimensional structure of the antiparallel G-quadruplex DL40. Although counterions are known to be stabilizing factors, the experimental structure of the DL40 used in this work appears to self-stabilize even without counterions. For this reason, we decided not to make arbitrary changes to the experimental model of the G-quadruplex DL40.

The ligand rises above the T4 and T20 base pair, forming a π-π stacking interaction and a cation-π interaction with the aromatic rings of T4 and T20, respectively. The T6 nucleotide lies alongside the ligand, resulting in a π-π T-shaped interaction, which contributes to the overall stability of the complex. Finally, the complex is further stabilized by a hydrogen bond between the OH group of the ligand and OP_2_T20 (2.68 Å).

## 4. Conclusions

In the framework of a project devoted to the identification of natural alkaloids that could selectively bind to G-quadruplex and i-motif structures, the interaction of escholidine with several guanine- and cytosine-rich sequences prone to form these structures has been studied. This interaction has been characterized by means of fluorescence titrations, melting experiments, and molecular modeling as well as NMR spectroscopy.

Fluorescence spectroscopy, as a highly sensitive method, enabled the detection of the interactions of escholidine with all studied G-quadruplex and i-motif structures through fluorescence quenching. The binding stoichiometry and stability constants were estimated from the fluorescence titration curves. Best fitting models were found to have stoichiometry, similarly to benzo[c]phenatridine alkaloids [40], 1:4 or 1:5. Since the fluorescence spectroscopy is a very sensitive method and can detect even weak changes in the fluorophore (alkaloid) environment; therefore, recorded changes in fluorescence intensities can also reflect non-specific interactions of alkaloid molecules to oligonucleotides.

The CD melting experiments have shown that alkaloid binding does not affect the thermal stability of i-motifs; therefore, interactions previously observed by fluorescence spectroscopy can be assigned as only weak electrostatic interactions.

On the other hand, a certain stabilization was observed for G-quadruplexes, specifically for the antiparallel structure adopted by DL40, being the difference in melting temperature 5.2 °C in this case. These results are in line with those previously published for alkaloids from the protoberberine group—berberine [44], corysamine, and coptisine [39].

NMR studies provided structural information on the interaction of escholidine with the considered sequences. Hence, for an interaction where the alkaloid does not fully intercalate into the G-quadruplex structure, and being the interaction most likely to go through the external loop, it causes an overall slight stabilization of the structure. Escholidine can be located over the external tetrads but covering only half of the guanine planes. This is proven by the shielding effect on the guanine resonance, which is lower than that of other ligands [38,39,40]. The interaction with DNA could be considered one of the mechanisms of action of these alkaloids, even if additional mechanisms involving other targets cannot be excluded.

The discrepancy between fluorescence titration experiments showing higher stoichiometry and other methods showing a 1:1 model can be explained by the strong interaction of one molecule of alkaloid to DNA (main) binding site and several weak and non-specific interactions of three to four alkaloid molecules to oligonucleotide surface. This non-specific interaction can be eventually assigned also as the stacking of alkaloids to each other when only the nearest alkaloid is attached to the oligonucleotide structure.

As the highest affinity to escholidine shows DL40 structure, the three-dimensional model of the complex between alkaloid and DL40 was prepared by means of molecular docking. The π-π stacking interaction and a cation-π interaction with the aromatic rings of T4 and T20, respectively, were found to be the most stabilizing factors accompanied by a π-π T-shaped interaction to the T6 nucleotide.

Concerning i-motif structures, NMR studies suggested some interaction of escholidine to the i-motif formed by nmyc01 with the disruption of the Watson–Crick base pairs of the internal hairpin structure. The same experiment with the mutated (myc01m) and 22nt sequences suggested that escholidine neither intercalated nor stacked externally to the cytosine bases. Hence, further research must be carried out to find natural alkaloids that could bind to i-motif structures.

## Figures and Tables

**Table 1:** Sequences of measured oligonucleotides.

Code	Sequence (5′–3′)	Type
GG1	GGGCGGGCGCGAGGGAGGGT	Parallel G-quadruplex [45] (pH 7)
DL40	GGTTTTGGCAGGGTTTTGGT	Antiparallel G-quadruplex [46] (pH 7)
nmyc01	ACCCCCTGCATCTGCATGCCCCCTCCCACCCCCT	i-motif + duplex hairpin [47] (pH 5)
nmyc01m	ACCCCCTGCATCTTTTTGCCCCCTCCCACCCCCT	I-motif [47] (pH 5)
22nt	CCCTAACCCTAACCCTAACCCT	I-motif [23] (pH 5)

**Table 2:** T_m_ values of pure oligonucleotides and mixtures.

Oligonucleotide	T_m_ [°C]		ΔT_m_ [°C]
	Pure	with ED	
GG1	48.1 ± 0.4	49.8 ± 1.4	1.7
DL40	28.8 ± 0.9	34.0 ± 0.8	5.2
nmyc01	58.1 ± 0.4	57.8 ± 1.6	−0.3
nmyc01m	55.8 ± 0.9	55.2 ± 1.1	−0.6
22nt	51.5 ± 0.1	52.2 ± 0.5	0.7

**Table 3:** Thermodynamic parameters for the folding process in the absence and presence of escholidine. These values were determined from the CD-monitored melting experiments assuming a two-state process and null constant values of the changes in enthalpy and entropy through the melting process. The associated uncertainties are 5% for ΔH and ΔS and 10% for ΔG.

Oligonucleotide	Pure			with ED		
	ΔG (37)	ΔH [kcal/mol]	ΔS [cal/K·mol]	ΔG (37)	ΔH [kcal/mol]	ΔS [cal/K·mol]
GG1	−1.4	−40.8	−127.1	−1.7	−42.5	−131.5
DL40	1.7	−62.3	−206.5	0.6	−56.2	−183.0
nmyc01	−5.4	−85.0	−256.7	−4.4	−70.1	−211.8
nmyc01m	−4.4	−77.9	−236.8	−3.8	−67.3	−205.0
22nt	−3.2	−71.4	−219.9	−3.7	−78.9	−242.4

**Table 4:** Log K_a_ constants and assumed stoichiometry for escholidine:DNA complex.

Oligonucleotide	Stoichiometry	Log K_a_
GG1	1:5	22.39 ± 0.03
DL40	1:4	18.46 ± 0.05
nmyc01	1:4	18.21 ± 0.17
nmyc01m	1:5	22.61 ± 0.08
22nt	1:4	17.64 ± 0.12

## Data Availability

Data sharing not applicable.

## Supplementary Materials

The following are available online at https://www.mdpi.com/article/10.3390/biology10121225/s1, Figure S1: CD melting curves of oligonucleotides. Table S1: Inter-residue NOE interactions of c-kit21T12T21 in the complex with escholidine, Table S2: Selected ^1^ H chemical shift values for the complex of escholidine with c-kit21T12T21, Figure S2: Example of a titration of escholidine with GG1. Table S3: Stoichimetry model, sum of squares and calculated logK values for interaction of escholidine with GG1. Figure S3: Dependence of the sum of squares of residuals vs. the DNA:ligand stoichiometry. Figure S4: Example of a titration of escholidine with DL_40. Figure S5: Example of a titration of escholidine with nmyc01. Figure S6: Example of a titration of escholidine with nmyc01m. Figure S7: Example of a titration of escholidine with 22nt.
