# Supplementary material for: Alkaloid Escholidine and Its Interaction with DNA Structures
Source: Biology (Basel). 2021 Nov 24;10(12):1225. doi: 10.3390/biology10121225 (PMC8698932; doi:10.3390/biology10121225)
Supplement: Supplementary file 1 [file biology-10-01225-s001.zip › biology-1390952-supplementary.pdf]

SUPPLEMENTARY

Alkaloid Escholidine and Its Interaction with DNA Structures

P. Jarošová[1], P. Hannig[1], K. Kolková[1], S. Mazzini[2], E. Táborská[3] R. Gargallo[4], G. Borgonovo[2], R. Artali[5] and P. Táborský[1*]

[1]Department of Chemistry, Faculty of Science, Masaryk University, Kamenice 5, 62500 Brno, Czech Republic

[2]Department of Food, Environmental and Nutritional Sciences (DEFENS), Section of Chemical and Biomolecular Sciences, University of Milan, Via Celoria 2, 20133 Milan, Italy

[3]Department of Biology, Faculty of Medicine, Masaryk University, Kamenice 5, 62500 Brno, Czech Republic

[4]Dept. of Chemical Engineering and Analytical Chemistry, University of Barcelona, Marti i Franquès 1, E-08028 Barcelona, Spain

[5] Scientia Advice di Roberto Artali, 20832 Desio, MB, Italy

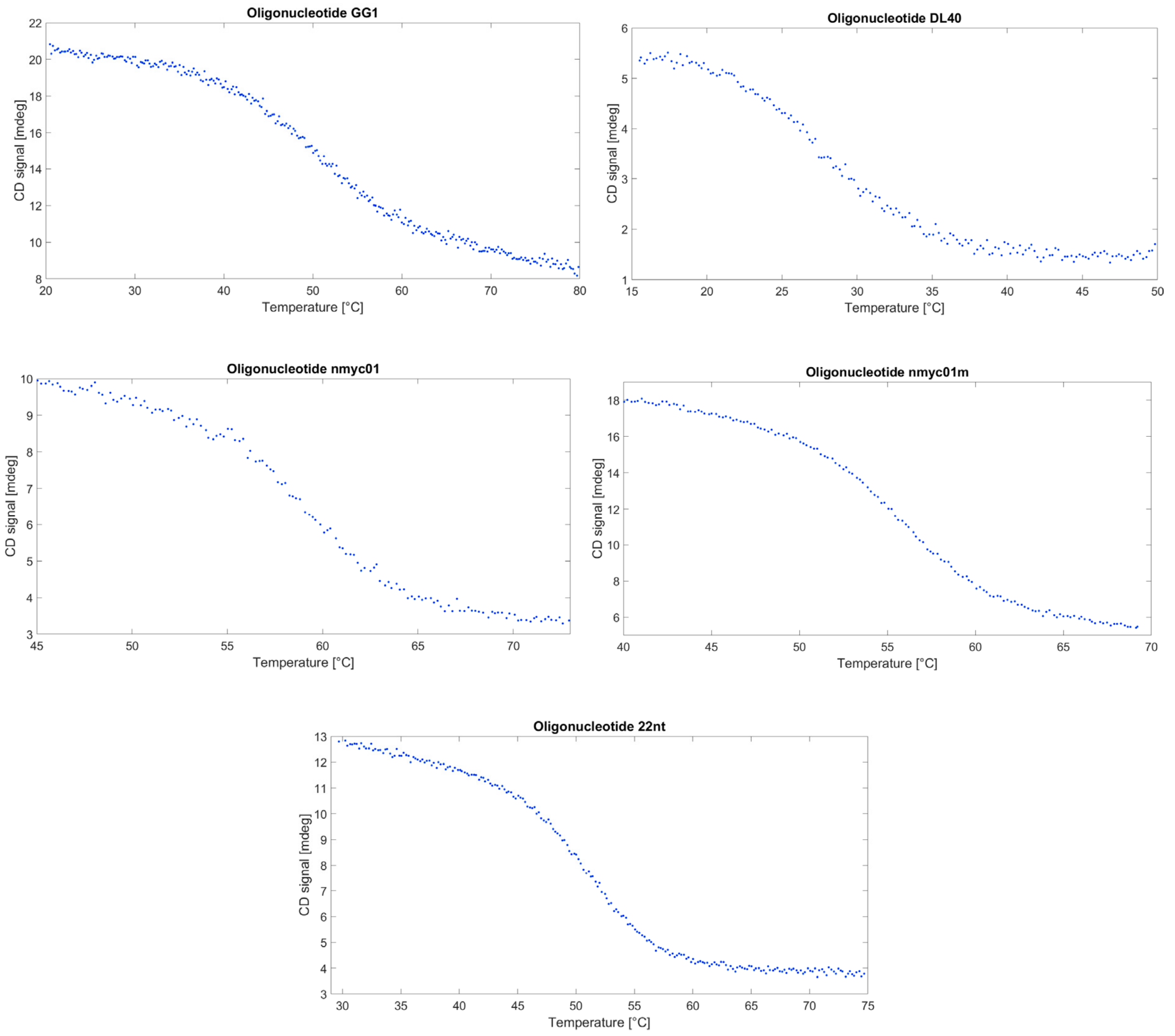

Figure S1. CD melting curves of oligonucleotides. (GG1 measured at 261 nm; DL40 at 291 nm; nmyc01, nmyc01m and 22nt at 287 nm).

Table S1. Inter-residue NOE interactions of c-kit21T12T21 in the complex with escholidine.

G-tetrad I	*G-tetrad II*	*Tetrad III*
G4H1…..G8H8	G3H1….G7H8	G18H1….G2H8
G8H1…G16H8	G7H1...G15H8	G14H1….G18H8
G16H1…G20H8	G15H1…G19H8	G6H1….G14H8
G20H1…G4H8	G19H1…G3H8	G2H1…..G6H8

Table S2. Selected ^1H chemical shift values for the complex of escholidine with c-kit21T12T21.[a]

c-kit	H1/H2/H5/CH_3	Δδ[c]	H6/H8	Δδ[c]
G2	11.79	**-0.19**	8.19	+0.04
G3	11.24	-0.08	8.22	+0.47
G4	10.92	**-0.22**	7.82	+0.12
G6	11.60	+0.04	7.95	-0.08
G7	11.49	-0.11	7.92	-0.06
G8	11.06	**-0.26**	7.91	+0.13
T12	1.74	-0.14		
A13	n.d.	-	8.34	+0.17
G14	11.82	-0.17	8.26	+0.01
G15	11.27	-0.13	7.88	+0.06
G16	10.92	-0.15	7.84	+0.07
A17	n.d.	-	8.54	+0.03
G18	11.68	**-0.14**	8.01	-0.01
G19	11.47	-0.11	7.99	-0.02
G20	11.10	**-0.18**	7.79	+0.12
T21	1.78	+0.32		

[a] Measured at 25°C in ppm (δ) from external DSS. Solvent H_2O-D_2O (90:10 v/v), 5 mM phosphate buffer, 20 mM KCl, pH 6.9. [b]R = 3.0. [c]$\Delta\delta = \delta_{bound} - \delta_{free}$

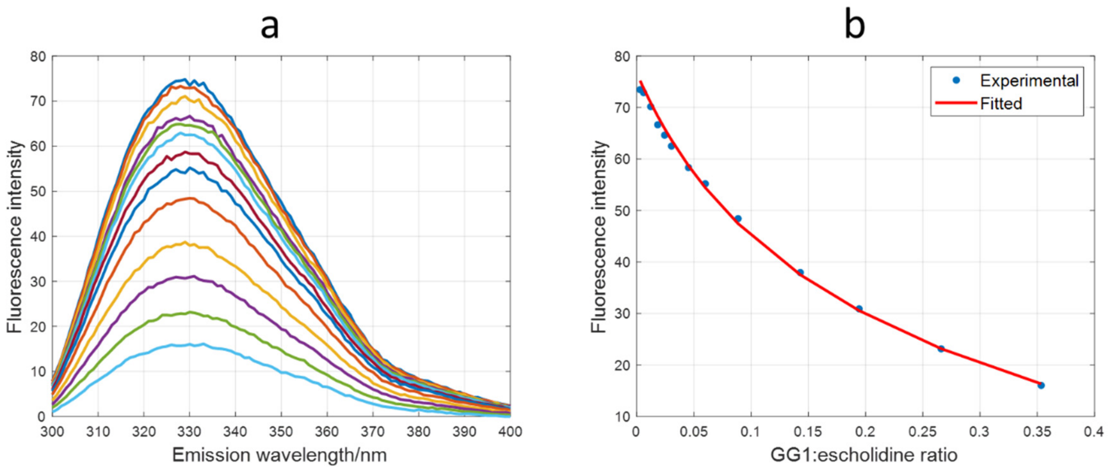

Figure S2. Example of a titration of escholidine with GG1. (left) Emission spectra recorded along the titration of escholidine with GG1. λ_{ex} = 285 nm), C_{esch} = 3 x 10^{-5} M, c_{DNA} varies from 0 (upper spectrum) to 1 x 10^{-5} M (lower spectrum). (right) Experimental and fitted fluorescence intensity measured at 330 nm as a function of GG1:escholidine ratio.

Table S3. Stoichimetry model, sum of squares and calculated logK values for interaction of escholidine with GG1.

1: X DNA:ligand stoichiomstry	Sum of squares	log K_a
1	15149.40	
2	5173.60	
3	641.69	
4	40.39	18.09
**5**	**35.59**	**22.25**
6	40.92	26.55
7	45.69	30.91

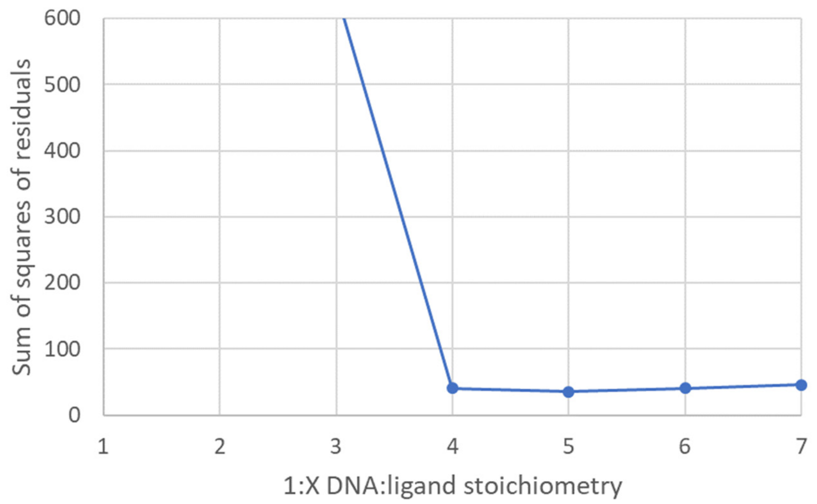

Figure S3. Dependence of the sum of squares of residuals *vs.* the DNA:ligand stoichiometry. The data correspond to one of the titrations of escholidine with GG1.

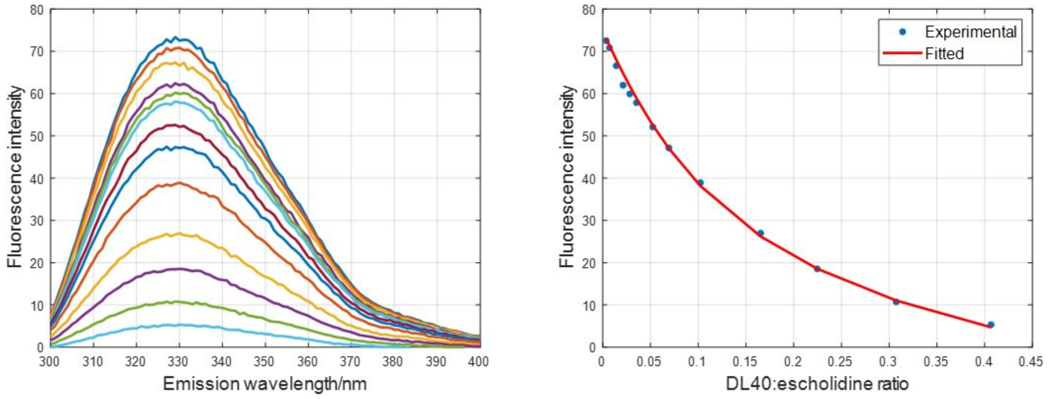

Figure S4. Example of a titration of escholidine with DL_40. (left) Emission spectra recorded along the titration of escholidine with GG1. λ_{ex} = 285 nm), C_{esch} = 3 x 10^{-5} M, c_{DNA} varies from 0 (upper spectrum) to 1 x 10^{-5} M (lower spectrum). (right) Experimental and fitted fluorescence intensity measured at 330 nm as a function of DL_40:escholidine ratio.

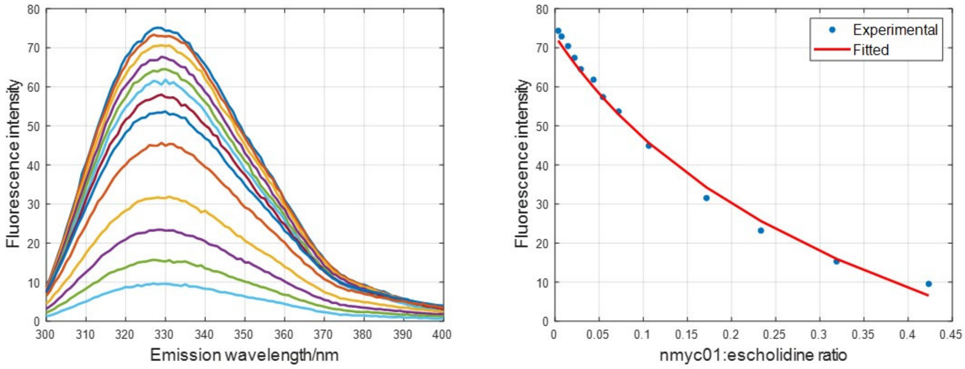

Figure S5. Example of a titration of escholidine with nmyc01. (left) Emission spectra recorded along the titration of escholidine with GG1. λ_{ex} = 285 nm), C_{esch} = 3 x 10^{-5} M, c_{DNA} varies from 0 (upper spectrum) to 1 x 10^{-5} M (lower spectrum). (right) Experimental and fitted fluorescence intensity measured at 330 nm as a function of nmyc01:escholidine ratio.

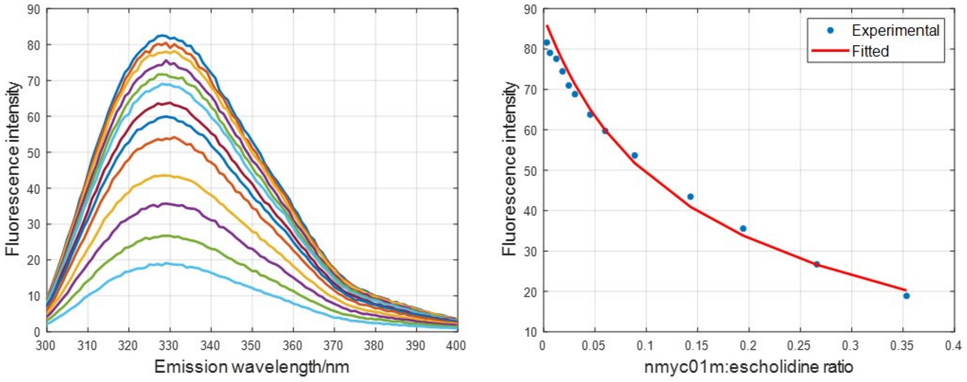

Figure S6. Example of a titration of escholidine with nmyc01m. (left) Emission spectra recorded along the titration of escholidine with GG1. λ_{ex} = 285 nm), C_{esch} = 3 x 10^{-5} M, c_{DNA} varies from 0 (upper spectrum) to 1 x 10^{-5} M (lower spectrum). (right) Experimental and fitted fluorescence intensity measured at 330 nm as a function of nmyc01m:escholidine ratio.

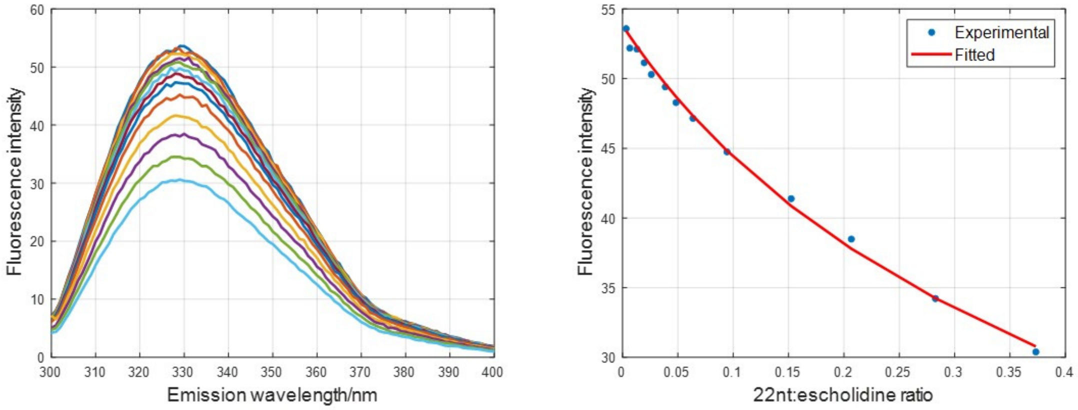

Figure S7. Example of a titration of escholidine with 22nt. (left) Emission spectra recorded along the titration of escholidine with GG1. λ_{ex} = 285 nm), C_{esch} = 3 x 10^{-5} M, c_{DNA} varies from 0 (upper spectrum) to 1 x 10^{-5} M (lower spectrum). (right) Experimental and fitted fluorescence intensity measured at 330 nm as a function of 22nt:escholidine ratio.
